# Can a human sing with an unseen artificial partner? Coordination dynamics when singing with an unseen human or artificial partner

**DOI:** 10.3389/frobt.2024.1463477

**Published:** 2024-12-09

**Authors:** Rina Nishiyama, Tetsushi Nonaka

**Affiliations:** Graduate School of Human Development and Environment, Kobe University, Kobe, Japan

**Keywords:** inter-personal coordination, anticipatory synchronization, strong anticipation, self-other integration, togetherness

## Abstract

This study investigated whether a singer’s coordination patterns differ when singing with an unseen human partner versus an unseen artificial partner (VOCALOID 6 voice synthesis software). We used cross-correlation analysis to compare the correlation of the amplitude envelope time series between the partner’s and the participant’s singing voices. We also conducted a Granger causality test to determine whether the past amplitude envelope of the partner helps predict the future amplitude envelope of the participants, or if the reverse is true. We found more pronounced characteristics of anticipatory synchronization and increased similarity in the unfolding dynamics of the amplitude envelopes in the human-partner condition compared to the artificial-partner condition, despite the tempo fluctuations in the human-partner condition. The results suggested that subtle qualities of the human singing voice, possibly stemming from intrinsic dynamics of the human body, may contain information that enables human agents to align their singing behavior dynamics with a human partner.

## 1 Introduction

Music is an inherently social activity that involves real-time non-verbal communication (Volpe et al., 2016; Welch et al., 2014). A substantial amount of research in music performance has concentrated on exploring what insights musical interaction can provide into general inter-agent interaction (e.g., Moran, 2014; Sawyer, 2014). The ability to synchronize among musicians involves perceiving another’s actions and then adjusting one’s own actions accordingly, which includes continuous micro-timing adjustments and anticipatory control (Proksch et al., 2022; Keller et al., 2007; Konvalinka et al., 2010; Sebanz et al., 2006). Therefore, interactions in music are regarded as valuable models for exploring complex inter-agent interaction dynamics (D’Ausilio et al., 2015), with potential implications for understanding coordination in human-machine interactions (Kim and André, 2008).

In recent decades, computer-based technologies have rapidly advanced in their ability to generate human-like acoustic features in speech, singing, and music playing (Umbert et al., 2015; Kühne et al., 2020). One example is singing voice synthesis technology, which enables computers to “sing” any song. Since the release of singing voice synthesis software VOCALOID, it has become especially popular in Japan (Kenmochi and Ohshita, 2007). Although technologies to mimic human-like acoustic features in music playing have rapidly improved, the social and collaborative implications of such music technology have remained unexplored. Little progress has been made in designing artificial partners that offer a comparable social experience to playing with another person, despite the fundamental importance of understanding what enables humans and machines to interact with each other in a coordinated manner.

Previous studies have shown that bodily movements are crucial for communication and expression among musicians (Klein et al., 2022), enabling temporal anticipation of actions to synchronize with others (Keller et al., 2007; Burger et al., 2014; Saino, 2019; Schreibelmayr and Mara, 2022). Chang et al. (2017) discovered that the body sway of one musician influenced that of others, even when visual contact between them was absent. Since visual contact was absent, body sway could not have directly conveyed information about the partner to them. It has been suggested that body sway may manifest in the sound produced by musicians, allowing them to coordinate their behaviors with each other by perceiving subtle auditory information during music performance (Klein et al., 2022). This raises the question: Can a musician coordinate with the sound produced by an artificial agent without a human body and still play music together in a coordinated manner?

The current study employs VOCALOID 6 (Yamaha Corporation, Hamamatsu, Japan), an AI-based software designed to generate natural-sounding and highly expressive singing voices from lyrics and melodies, to explore the following question: When a human singer performs a song with an unseen artificial partner versus an unseen human partner, will the degree of inter-agent synchrony and the similarity of their singing voices’ amplitude envelopes differ between these two conditions? This test examines whether the coordination dynamics between a singer and an unseen partner are indistinguishable when the partner is either human or artificial (c.f., Dotov et al., 2024).

In the present study, the artificial partner (Vocaloid) maintained a consistent tempo throughout the musical passage. In contrast, the human partner sang at her own tempo after listening to a two-bar metronome cue set at the same tempo as the artificial partner. Intuitively, it might seem easier for a singer to synchronize with music that maintains a constant tempo. However, if real human singing voices convey subtle information about how dynamics unfold at different time scales, then the small fluctuations in a human voice may not be a hindrance. On the contrary, these variations could provide prospective information that aids in singing along with the voice. If this latter scenario holds true, we would expect a higher degree of inter-agent synchrony and dynamic similarity when a singer performs with a human partner compared to when they sing with an artificial partner. Moreover, if the fluctuations in the human singing voice contain prospective information about how the music unfolds, then stronger anticipatory adjustments of a singer in such a way to tune into the dynamics of a partner would be expected in the condition where a singer sings along with a human partner compared to the condition involving an artificial partner.

We tested these hypotheses by conducting cross-correlation (CC) and Granger causality (GC) analyses on pairs of time series representing the amplitude envelopes of participants’ and their partners’ singing voices. CC measures the correlation between the two time series across time-lagged copies of one another within a range of positive and negative lags. The sign of the lag at which the largest CC value occurs suggests the temporal precedence between the two time series. Although CC indicates how similar the two time series are, it can also provide a measure of synchrony by quantifying the correlations with a zero lag when the two time series are positioned together in time (Klein et al., 2022). Granger causality is a statistical notion for time series data, where a preceding time series helps predict a future one (Barnett and Seth, 2014). Intuitively, if a signal is predictable, it is possible to make predictions about its future based on its past history. If the amplitude envelopes of two vocalists singing together are denoted as *Y*(*t*) and *X*(*t*), and they are correlated, then the past of *X* can also be used to predict the future of *Y*. If the inclusion of *X* improves the prediction of future of *Y*, then *Y* is considered to be Granger-caused by *X* (for details, see Barnett and Seth, 2014). The Granger causality (GC) test can also reveal anticipatory dynamics, where the follower displays behavior that precedes that of its leader in time (Chen et al., 2023).

In the present study, first, we expected higher maximum CC values to indicate stronger correlation between dynamics of singing voices between a singer and its partner. Second, we expected a higher zero-lag CC values to indicate higher synchrony between dynamics of singing voices between a singer and its partner. Third, we expected that the lag at which the highest cross-correlation (CC) value occurs, along with the directional difference in Granger causality (from partner to participant versus from participant to partner), would indicate the extent of anticipatory adjustments made while singing with a partner.

## 2 Methods

### 2.1 Participants

Ethical approval for the study (# 725–2) was obtained from the Ethics Committee at the Graduate School of Human Development and Environment, Kobe University (Japan). We recruited seven university students. Because a female synthesized voice was used in the experiment (see the following section), all the participants we recruited were female. All participants had experience singing in a group setting, such as in a school choir. The average age of the participants at the time of the experiment was 22.9 (SD = 0.8). In addition, we hired a female semi-professional female singer who had been singing popular music in a local band to record a human singing voice to be used as a human partner, with which participants were instructed to sing along. No participants reported having hearing or motor disability. Participants received a small fee for their participation.

### 2.2 Music materials

**FIGURE 1:**
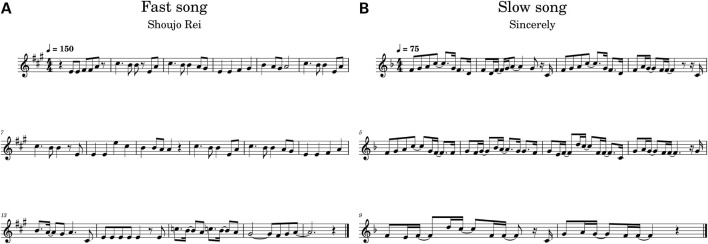
The refrains of the two songs sung in the experiment: **(A)** Slow Song (“Sincerely” originally sung by *TRUE* and **(B)** Fast Song (“Shoujo Rei” originally sung by *Miku Hatsune*).

We selected two Japanese popular songs with different tempi, *Sincerely* (75 BPM in 4/4 time, hereafter referred to as a “slow song”) and *Shojo Rei* (Tempo: 150 BPM in 4/4 time, hereafter referred to as a “fast song”), for participants to sing along (Figure 1). Both songs were sung by a female vocalist in their original versions. Slow Song was originally sung by a human female artist *TRUE*, while Fast Song was originally sung by a female VOCALOID virtual singer Miku Hatsune. Fast Song involves a series of notes that are an octave (8 notes) apart, while Slow song has a relatively simple melody with smaller note changes (Figure 1). The chorus/refrain part was extracted from each song to be sung by participants in the experiment (Slow Song: 10 bars, 37 s, Fast Song: 17 bars, 29 s).

For each song, we created an audio recording with a human singer and a VOCALOID AI singer (HARUKA), which was used as a singing voice of a partner for participants to sing along. First, recordings of an artificial Vocaloid singer were created with VOCALOID 6 singing voice synthesis software (audio files are available in Supplementary Material). Next, we sent the audio recordings of a Vocaloid singer to a human singer and instructed her to sing as close to the vocal style and pitch changes of the Vocaloid singer as possible (audio files are available in Supplementary Material). Both singing voices were recorded in Cubase LE AI Elements 13 (Steinberg, Hamburg, Germany) as monophonic 16-bitWAV files at 44.1 kHz sampling rate. The recording of both songs included the two measures of metronome sounds before the chorus began. Once the chorus has begun, no metronome clicks were available for listeners to hear in either partner condition. In other words, the pickup metronome clicks indicated only the starting tempo of the pieces. While the Vocaloid partner sung with a constant tempo throughout the chorus, the human partner sung with her own tempo after listening to the two-bar metronome sounds which indicated the beginning of the recording. The vocal recordings of both the Vocaloid partner and the human partner are available in Supplementary Material.

### 2.3 Procedure

**FIGURE 2:**
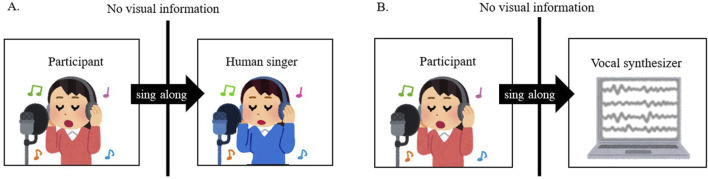
Experimental setup. **(A)** Human partner condition where participants sing along with a human partner. **(B)** Artificial partner condition where participants sing along with the vocal synthesizer.

Chorus parts for both pieces sung by an artificial (Vocaloid) singer and a human singer were sent to participants a week prior to the experiments for participants. Each participant was instructed to sing along in unison with the singing voice of an unseen partner (either a human or an artificial partner) played through a headphone monitor, following the partner’s singing voice as closely as possible (Figure 2). The experimental setting was analogous to the situation where a singer sings along with a partner in a separate room without visual information. Before the start of the singing voice, participants heard the two-bar metronome (as was the case in the recording of the singing voice of the human partner) so that they can adjust the timing at the beginning. The singing voice of each participant was recorded on Cubase LE AI Elements 13 with Scarlett 2i2 Studio microphone, pre-amp, and USB audio interface (Focusrite, High Wycombe, United Kingdom). When each participant arrived in the lab, participants practiced singing along with the recordings for a few times to test participants’ recording setups to familiarize them with the procedure. Participants recorded five times for each song (Slow Song and Fast Song) in each partner condition (a human partner and an artificial partner). All participants sang Slow Song first before singing Fast Song. Within each song, the order of the trials of the two partner conditions (a human partner vs. an artificial partner) was randomized.

### 2.4 Data analysis

#### 2.4.1 Amplitude envelope

All vocal audio recordings were imported into MATLAB R2023b (MathWorks, Natick, MA) and resampled to a rate of 16 kHz. The temporal envelope of each vocal sample was obtained by computing the magnitude of the Hilbert transform (Issa et al., 2024; Braiman et al., 2018). Building on previous studies that analyzed temporal modulations in human speech and music separately from spectral information (Ding et al., 2017; Elliott and Theunissen, 2009), we isolated slow temporal modulations in sound amplitude (<32 Hz) by applying a 3rd order Butterworth IIR filter with a 32 Hz cutoff frequency to the time series twice—once forward and once backward. The amplitude envelope time series were then down-sampled to 200 Hz by averaging the time points within consecutive, non-overlapping 5-ms windows. This process resulted in a smooth curve that reflects the fluctuations in sound intensity over time.

#### 2.4.2 Cross correlation

To assess the similarity of temporal modulations in sound intensity between participants’ performances and the recorded vocal sounds of their partners that the participants followed, we calculated cross-correlations (CC) between the amplitude envelope time series of each partner’s recording (a human singer and a Vocaloid singer) and each participant’s performance during every trial while singing along in unison. CC coefficients were calculated across the entire waveforms for each trial for lags between −0.1 and 0.1 s.

We derived three measures based on the CC analysis:

1. Maximum CC Value: The highest CC coefficient across all time lags was calculated for each trial, resulting in one cross-correlation coefficient per trial per participant. This value indicates the similarity of the amplitude envelope time series between the partner’s and the participant’s voices.
2. Lag at Maximum Correlation: The time delay at which the maximum correlation occurred in each trial was identified. This measure indicates the time difference between the two time series that produced the highest similarity. Positive lag values suggest that the participant’s voice lagged behind the partner’s voice, while negative lag values indicate that the participant’s voice preceded the partner’s.
3. Zero Lag CC Coefficient: The CC coefficients were computed with a zero lag, where the two time series are aligned in time. This measure indicates the phase alignment synchrony between the amplitude envelopes of the partner’s and the participant’s singing voices.

#### 2.4.3 Granger causality

We calculated the magnitude of Granger causality (GC) from the amplitude envelope time series of the recording of the partner (Human or Vocaloid) to the amplitude envelope time series of the performance of the participant and vice versa—for each participant and each trial following the procedure implemented in the Multivariate Granger Causality (MVGC) Toolbox for MATLAB (Barnett and Seth, 2014). An optimal model order (the number of past points in the time series included in the model) was chosen for each trial for each participant using the Akaike information criterion. Then, for each participant for each song, the maximum model order out of their five trials was used to calculate GC values for all five trials. The average model orders used for participants when singing Fast Song and Slow Song were 75 ms (15 points) and 85 ms (17 points), respectively.

#### 2.4.4 Statistical analysis

We modeled the three aforementioned CC measures as outcome variables in a linear mixed effects model using the “lme” function from the “nlme” package (Pinheiro et al., 2024) in R version 4.4.1. The fixed effects factors were partner (a human singer vs. an artificial Vocaloid singer), and tempo (Slow Song vs. Fast Song). Similarly, we modeled GC as an outcome variable in a linear mixed effects model with partner, tempo, and the direction of GC (partner to participant vs. participant to partner) as fixed effects. To account for the correlation between performances of the same participant, a participant factor was included as a random effect for the intercept for both models. Bonferroni-adjusted *post hoc* pairwise comparisons based on estimated marginal means were conducted using the “emmeans” package (Lenth, 2024) in R. The alpha value for a significant effect was set at 0.05.

## 3 Results

**FIGURE 3:**
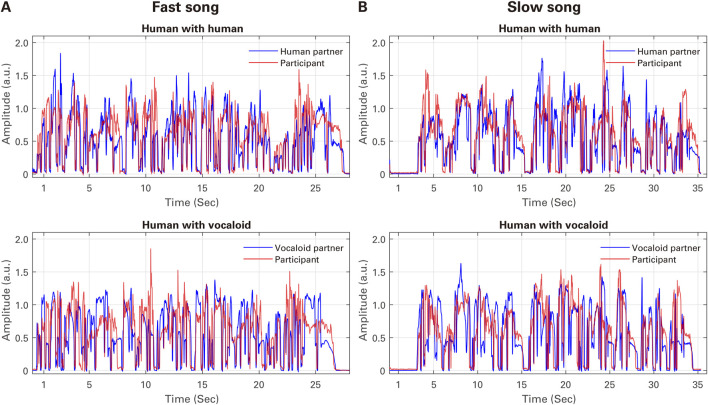
Examples of the audio amplitude envelope time of the singing voice in the two partner conditions (a human partner and an artificial Vocaloid partner) for **(A)** Fast Song and **(B)** Slow Song from the experiment (each taken from the fifth trial of participant ID P5).

Figure 3 presents the examples of the audio amplitude envelope time series of the singing voice in the two partner conditions (a human partner and an artificial Vocaloid partner) for (A) Fast Song and (B) Slow Song from the experiment. Visual inspection of the time series suggests that the singing voice of the participant generally closely mirrored that of the partner in both partner conditions and songs with different tempi.

**FIGURE 4:**
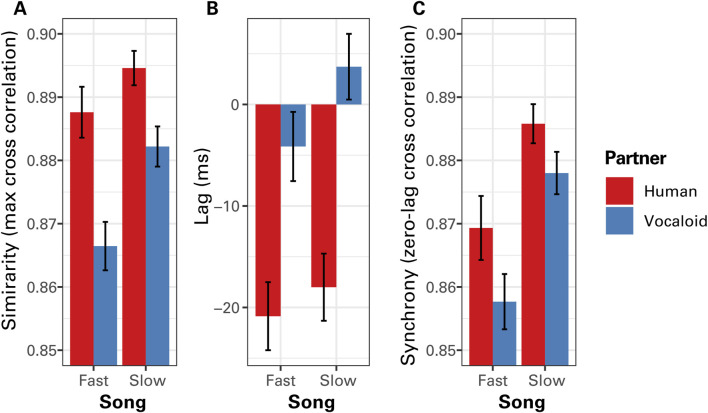
The results of cross correlation (CC) analysis of the temporal modulations in sound amplitude between the partner and the participant as a function of tempo (Fast Song vs. Slow Song) and the type of partner (Human or Vocaloid). **(A)** The maximum values of the CC coefficients across all time lags that indicate the similarity of the amplitude envelope time series between the partner and the participant. **(B)** The lag at which the maximum correlation occurred, which indicates the time delay between the two time series that produced the highest degree of similarity. **(C)** Cross correlation coefficients computed with a zero lag when the two time series are positioned together in time that indicate a measure of phase alignment between the singing voices of the partner and the participant. Positive lag values would indicate that the singing voice of the participant lags behind that of the singing voice of the partner, where lagging the voice of the partner produced the highest degree of similarity, while negative lag values would indicate that the singing voices of the participant preceded that of the partner. Error bars representu ±1 standard error of the mean.

Figure 4 presents the results of the cross-correlation analysis when the data from all participants were pooled. Visual inspection of Figure 4A suggests that when a participant sung along with an unseen partner in unison, the participant generally exhibited greater degree of similarity of the temporal modulation of sound intensity with the human partner compared to the artificial Vocaloid partner, and in the Slow Song compared to the Fast Song. A linear mixed effect model analysis on maximum CC coefficients confirmed these impressions, finding a highly significant effect of partner, *F*_(1,130)_ = 91.30, *p* < 0.0001. The linear mixed effect model analysis also found a significant effect of tempo, *F*_(1,130)_ = 41.87, *p* < 0.0001, with a relatively weak significant interaction between partner and tempo, *F*_(1,130)_ = 6.22, *p* = 0.014, where the difference in the degree of similarity between the human and the Vocaloid partners was greater when participants sang the Fast Song compared to the Slow Song. No other significant effects were found. A Bonferroni-corrected *post hoc* analysis confirmed that the participant exhibited a significantly greater degree of similarity of the temporal modulation of sound intensity with the human partner compared to the artificial Vocaloid partner when singing Fast Song, *t* (130) = 8.52, *p* < 0.0001, as well as when singing Slow Song, *t* (130) = 4.99, *p* < 0.0001.

The phase relations between the singing voice of participants and that of the unseen partner exhibited a difference between the human partner condition and the artificial Vocaloid partner condition. When a participant sang along with a human partner, she generally coordinated with the partner in an anticipatory manner, preceding the partner (Figure 4B). By contrast, when participants sung along with an unseen artificial partner, participants did not go ahead of the partner as much. A linear mixed effects model analysis on the lag at which maximum correlations occurred found a significant effect of partner, *F*_(1,130)_ = 184.62, *p* < 0.0001, confirming the above impression. The analysis also found a significant effect of tempo, *F*_(1,130)_ = 14.35, *p* = 0.0002, where the participants got ahead of the partner to a greater degree when singing the Fast Song compared to the Slow Song. No other significant effects were found.

A similar tendency was observed in the zero-lag cross correlation values which indicate the degree of synchrony of the amplitude envelope time series between voices of the partner and the participant (Figure 4C). A linear mixed-effects model on zero-lag cross-correlation (CC) coefficients revealed a significant effect of partner type, *F*_(1,130)_ = 16.62, *p* = 0.0001, indicating that participants in this experiment were more synchronized with the unseen human partner than with the artificial Vocaloid partner. This finding is somewhat counterintuitive, given that the Vocaloid partner maintained a constant tempo, whereas the human partner did not. The analysis also found that participants were generally more synchronized with the partner when they were singing the Slow Song compared to the Fast Song, *F*_(1,130)_ = 59.52, *p* < 0.0001. No other significant effects were found.

**FIGURE 5:**
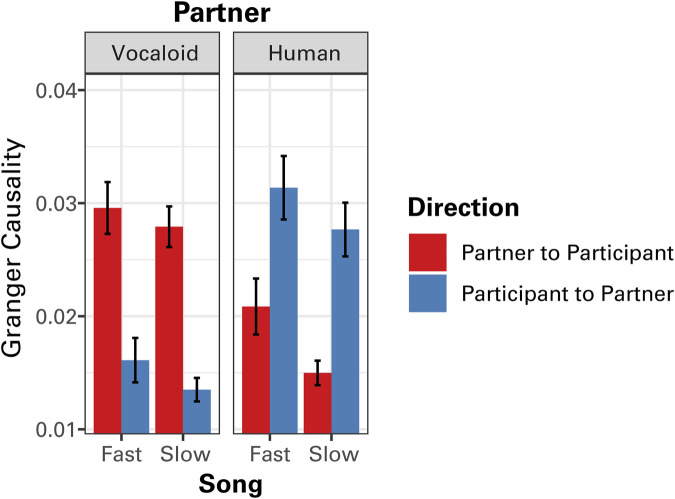
The results of Granger causality (GC) analysis of the temporal modulations in sound amplitude between the partner and the participant as a function of tempo (Fast Song vs. Slow Song) and the type of partner (Human or Vocaloid). Granger causality values for both directions, from the amplitude envelope of the partner to that of the participant, and from the amplitude envelope of the participant to that of the partner are presented. Error bars represent ±1 standard error of the mean.

Figure 5 presents the GC values in the two directions (partner to participants; participants to partner) within-subjects for each tempo separately. When participants sang along with the Vocaloid partner, as expected, participants generally followed the partner because the recording of the partner was fixed. However, with the human partner, the tendency reversed, where participants exhibited the anticipatory dynamics leading the human partner (Figure 5). A linear mixed effect effects model analysis on GC values confirmed the visual impression, finding a highly significant interaction between partner and direction, *F*_(1,266)_ = 86.65, *p* < 0.0001. This result confirmed the presence of anticipatory dynamics when singing with the human partner, with participants displaying the temporal modulation of sound intensity that preceded their partner’s in time. It also indicated that, when singing with the Vocaloid partner, participants generally followed the partner’s lead. In addition, the analysis found a marginally significant effect of tempo, *F*_(1,266)_ = 6.35, *p* = 0.012, where GC values tended to be greater in the Fast Song compared to the Slow Song. No other significant effects were found.

**FIGURE 6:**
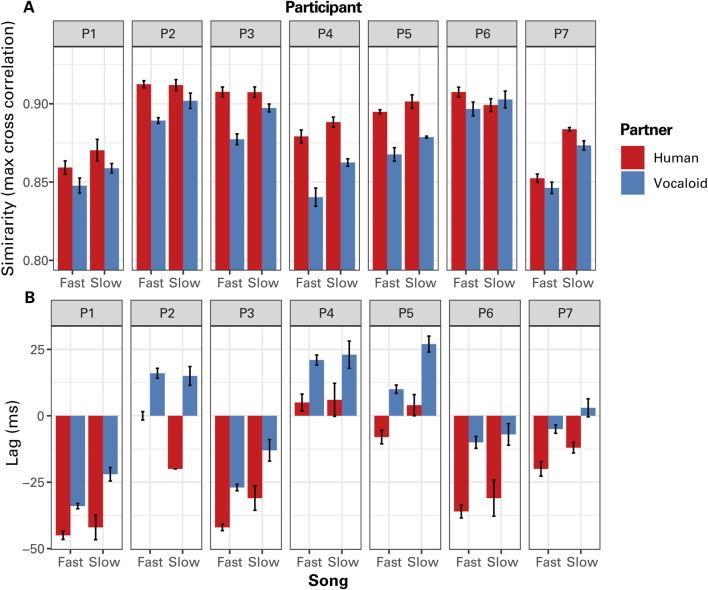
The results of cross correlation (CC) analysis of the temporal modulations in sound amplitude between the partner and the participant as a function of tempo (Fast Song vs. Slow Song) and the type of partner (Human or Vocaloid) for individual participants (P1 - P7). **(A)** The maximum values of the CC coefficients, and **(B)** the lag at which the maximum correlation occurred.

The data from individual participants demonstrated that the participants generally sang with more similar temporal variation patterns with the unseen human partner compared to the artificial Vocaloid partner, except one individual (P6) who did not exhibit such a difference when singing the Slow Song (Figure 6).

**FIGURE 7:**
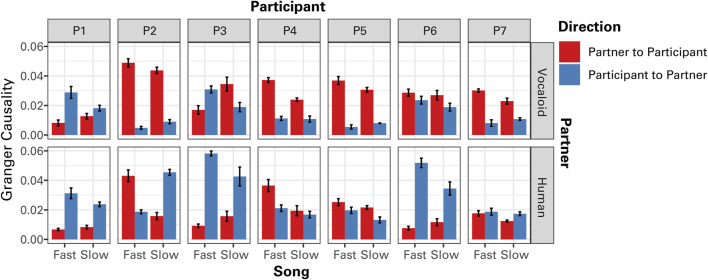
The results of Granger causality (GC) analysis of the temporal modulations in sound amplitude between the partner and the participant as a function of tempo (Fast Song vs. Slow Song) and the type of partner (Human or Vocaloid) for individual participants (P1 - P7). Granger causality values for both directions, from the amplitude envelope of the partner to that of the participant, and from the amplitude envelope of the participant to that of the partner are presented. Error bars represent ±1 standard error of the mean.

Individual GC data indicated that some participants consistently followed (P4 and P5) or led (P1) their partner across both conditions (Figure 7). Nevertheless, all participants exhibited a stronger tendency to synchronize in an anticipatory manner with the unseen human partner than with the artificial Vocaloid partner, though the degree of this effect varied among them (Figure 7).

## 4 Discussion

Music, typically involving multiple agents engaged in a temporally coherent manner, provides an ideal platform for studying the dynamics of human interaction with artificial agents. The primary objective of this study was to explore whether a human singer can effectively “sing together” with an unseen partner—a human singer or a vocal synthesizer—necessitating the synchronization of one’s behavior with the dynamic singing patterns of the partner in an anticipatory manner. We specifically investigated whether a human singer demonstrates similar time-varying properties in their singing voice when singing alongside two different partners: another human singer and a Vocaloid singer. We investigated whether the temporal correlations between a human singer’s amplitude envelope and that of an unseen partner could be distinguished across conditions featuring a human partner versus an artificial Vocaloid partner.

The results of the present experiment suggested that participants in the present experiment distinguished the human partner and the artificial partner by “doing.” Even without visual cues, our participants synchronized their singing behavior more anticipatorily with the human partner compared to the artificial partner, highlighting distinct dynamics between the two partners. This anticipatory synchronization led to a closer temporal alignment of the amplitude envelopes of singing sounds with those of the human partner compared to the artificial partner condition.

Considering the subtle tempo fluctuations in the human partner’s singing voice, it is unlikely that our participants relied on a “constant tempo” strategy, where they would maintain a steady tempo akin to a metronome in their minds. What alternative strategies enable human participants to better coordinate their singing voice with a fluctuating tempo compared to a constant tempo? One possible interpretation of the present results is that the singing sound of the human partner contained detectable information related to the unfolding of its dynamics in the near future (e.g., breath sounds). If perceptually detectable information exists in the human voice (but not in the voice of Vocaloid) that enables the coupling of the present change of state to the upcoming state of affairs, then anticipatory synchronization through perceptual coupling to auditory information is theoretically feasible (Stepp and Turvey, 2010). If a human participant successfully tunes into such information about the near future available in the present (termed as the “current future” in Bootsma, 2009), then we would expect to observe two key results from the present experiment: a pronounced tendency for anticipation and an increased similarity in the unfolding dynamics of the amplitude envelopes in the human-partner condition compared to the artificial partner condition.

Another plausible explanation for these results is that human participants and their human partners share similar intrinsic dynamics of the body. Consequently, singing with a human singer is naturally more coordinated compared to singing with a Vocaloid singer, which lacks these shared intrinsic dynamics. The dynamics of the singing voice are influenced by the air pressure in the lungs and the mechanical properties of the elastic folds of the mucous membrane lining the larynx (vocal cords), which are controlled by numerous laryngeal muscles (Sundberg, 1977). Considering the intricate biomechanics involved in singing, the dynamic unfolding of the singing voice is expected to reflect the characteristics of human vocal organs operating within specific biomechanical and environmental constraints. Therefore, it is likely that the amplitude envelopes of singing voices from human participants and their human partners would naturally align, displaying similar patterns of temporal unfolding. Furthermore, it is plausible that shared intrinsic dynamics enable the singer to attune to subtle, detectable information about the unfolding of the partner’s singing voice. The present findings suggest that participants found it easier to sing along with the human partner than with the artificial partner. Because human singers share the intrinsic dynamics of the body, they may naturally synchronize their rhythm with that of a partner when subtle auditory information about the partner’s embodied action is available. While some intrinsic dynamics are universally shared (e.g., airflow through the vocal folds is essential for singing), social or normative aspects of communal singing practices might also influence the degree of temporal alignment between two human singers (see Nonaka et al., 2024, for a related discussion in pottery practice). Future research could investigate how a shared community of practice impacts temporal alignment between singers when they sing together.

Future research could explore identifying auditory variables that enable a human singer to coordinate effectively with others. Once these variables are identified, they could be implemented in vocal synthesizers to experiment with manipulating these variables and observing how such changes affect the dynamics of singing together. Another intriguing area of research could be to explore the nature of morphological computation involved in singing (c.f., Pfeifer et al., 2007). By carefully considering the biomechanics and passive dynamics that create the distinctive patterns of human singing, it may be possible to replicate these temporal patterns so precisely that human participants would find the behavior of human and artificial partners indistinguishable.

## 5 Conclusion

The present study tested whether a singer’s coordination patterns differ when singing along with an unseen human partner versus an artificial partner (VOCALOID 6 voice synthesis software). We used cross-correlation analysis to compare the similarity of the amplitude envelope time series between the partner’s and participant’s voices, the time delay that produced the highest degree of similarity between the two time series, and the degree of phase alignment (i.e., synchrony) between the amplitude envelopes of the partner’s and participant’s singing voices. We also conducted a Granger causality test to determine whether the past amplitude envelope of the partner helps predict the future amplitude envelope of the participants, or if the reverse is true. We observed more pronounced anticipatory synchronization and greater similarity in the unfolding dynamics of amplitude envelopes in the human-partner condition compared to the artificial partner condition, despite the tempo fluctuations in the human-partner condition. The results suggest that subtle qualities of the human singing voice, possibly stemming from the intrinsic dynamics of the human body, may provide information that enables human singers to align their singing behavior with that of a human partner.

## Data Availability

The raw data supporting the conclusions of this article are available on request from the corresponding author.
